# Naringin Suppresses CoCl_2_-Induced Ferroptosis in ARPE-19 Cells

**DOI:** 10.3390/antiox14020236

**Published:** 2025-02-18

**Authors:** Yuchang Yang, Manting Liu, Xiaoxv Dong, Jie Bai, Wenjuan Shi, Qian Zhu, Juan Liu, Ziheng Wang, Lisa Yi, Xingbin Yin, Jian Ni, Changhai Qu

**Affiliations:** College of Chinese Herbal Medicine, Beijing University of Chinese Medicine, Beijing 102488, China; 20240941529@bucm.edu.cn (Y.Y.); 20230941503@bucm.edu.cn (M.L.); 201801020@bucm.edu.cn (X.D.); 20190935127@bucm.edu.cn (J.B.); 20220935136@bucm.edu.cn (Q.Z.); 20230935146@bucm.edu.cn (J.L.); 20240935163@bucm.edu.cn (Z.W.); 20240935162@bucm.edu.cn (L.Y.); yxbtcm@bucm.edu.cn (X.Y.)

**Keywords:** naringin, ARPE-19, branch retinal vein occlusion, ferroptosis, neovascularizat1ion

## Abstract

Hypoxic damage to retinal pigment epithelial (RPE) cells and subsequent neovascularization are key factors in the pathogenesis of branch retinal vein occlusion (BRVO). Naringin (NG), a naturally occurring flavanone glycoside, has demonstrated significant antioxidant and anti-neovascular activities. However, the regulatory effects and mechanisms of NG on ferroptosis in BRVO are yet to be explored. Our study aimed to investigate the protective effects of NG on RPE cells under hypoxic stress and to elucidate the underlying molecular mechanisms. Our findings revealed that NG significantly reduced cytotoxicity induced by cobaltous chloride (CoCl_2_) and also inhibited vascular proliferation in the retina, thereby attenuating choroidal neovascularization. NG pretreatment largely countered the overproduction of reactive oxygen species (ROS) and malondialdehyde (MDA) triggered by hypoxic damage, while also restoring levels of the antioxidants glutathione (GSH) and superoxide dismutase (SOD). Furthermore, NG pretreatment significantly activated the expression of hypoxia-inducible factor-1 alpha (HIF-1α) and its downstream heme oxygenase-1 (HO-1) and NADPH dehydrogenase (NQO1). In conclusion, NG not only inhibits neovascularization but also alleviates inflammation in RPE cells by modulating the HO-1/GPX4 pathway to inhibit ferroptosis. These findings highlight the potential of NG as a promising therapeutic agent for the treatment of BRVO.

## 1. Introduction

Branch retinal vein occlusion (BRVO), a significant cause of irreversible vision loss, ranks among the most prevalent retinal vascular disorders in adults [1,2]. A frequent complication of BRVO, neovascularization, poses a serious threat to vision, often leading to severe impairment [3]. Retinal pigment epithelial (RPE) cells form a monolayer of metabolically active cells located on Bruch’s membrane, situated between the neurosensory retina and the vascular choroid [4]. These cells play a critical role in maintaining retinal health and supporting photoreceptor function [5].

The primary pathological changes associated with BRVO involve the disruption of the blood-retinal barrier (BRB) due to damage to RPE cells, as well as neovascularization [6]. As structural changes in the BRB contribute to hypoxia and dysregulation, along with the changes in its surrounding microenvironment, including hypoxia and inflammation, they are found to be associated with BRVO. As a result, understanding the mechanisms of RPE cell damage is crucial for unraveling BRVO pathology and advancing the development of novel therapeutic strategies. The activity of RPE cells is heavily reliant on an adequate energy supply, resulting in high reactive oxygen species (ROS) production [7]. Under normal circumstances, the cellular antioxidant system effectively neutralizes excessive ROS. However, in the case of BRVO, ROS production surges due to compromised antioxidant system functionality. Hypoxia, a primary contributing factor, further exacerbates the situation by diminishing the vitality and function of RPE cells [8]. Many studies have investigated RPE cell death mechanisms by inducing oxidative stress. Hypoxia triggers the stabilization of hypoxia-inducible factors (HIFs), which are cellular oxygen sensors that facilitate adaptation to hypoxic conditions by regulating many oxygen-dependent genes [9]. HIFs are protective in the short term, but chronic hypoxia, as observed in BRVO, can be detrimental due to HIF-dependent inflammation, neovascularization, metabolic shift, and impaired lipid transport [10]. Histopathological studies of BRVO suggest that hypoxia is accompanied by oxidative stress. Therefore, an in vitro RPE model combining oxidative stress and hypoxia would mimic the conditions observed in BRVO. Cobalt chloride (CoCl_2_) can substitute for ferrous ions in heme, causing structural alterations in the heme protein and impairing its function as an O_2_ sensor [11]. This, in turn, induces hypoxia and triggers DNA damage [12]. CoCl_2_ has been widely used as a hypoxia mimic in both in vivo and in vitro studies. Additionally, it amplifies ROS production in cells, exacerbating oxidative stress [13].

Ferroptosis, a novel form of cell death, is characterized by oxidative stress-induced ROS production and lipid peroxidation [14,15,16]. Recent studies have linked ferroptosis to the pathogenesis of eye diseases, including BRVO [17]. Notably, ferroptosis has been identified as a primary mechanism of hypoxia-induced cell death in retinal pigment epithelial (RPE) cells [18,19]. Thus, targeting ferroptosis pharmacologically has emerged as a promising therapeutic strategy for BRVO. Ferroptosis is characterized by the accumulation of lipid peroxides and ROS. Biochemically, heme oxygenase-1 (HO-1) and the intracellular glutamate/cystine antiporter system (Xc-system) are inhibited, glutathione (GSH) is depleted, and glutathione peroxidase 4 (GPX4) activity is reduced, leading to the accumulation of lipid peroxides and ROS, thereby promoting ferroptosis [20]. The Xc-system is a heterodimer composed of the light chain SLC7A11 (xCT) and the heavy chain SLC3A2 (4F2hc), which transports glutamate out of the cell while simultaneously importing cystine, which is involved in the synthesis of GSH [21]. GPX4 is the primary scavenger of intracellular lipid peroxides, converting GSH to oxidized glutathione while reducing toxic peroxides, making GPX4 a master regulator of ferroptosis [22].

In recent years, traditional Chinese medicine (TCM) has drawn considerable attention for its potential role in the prevention and treatment of BRVO [23,24,25]. Naringin (NG), a flavonoid with diverse biological properties, is widely present within citrus fruits [26]. It exhibits multiple beneficial activities, including anti-inflammatory, anti-neovascular, and antioxidant effects [27,28,29]. NG has the ability to inhibit the Fenton reaction of iron-ATP, possibly due to the presence of 4-ketone and 5-hydroxyl regions in its chemical structure, which facilitate iron chelation [30]. While the mechanisms underlying therapeutic effects on various diseases have become clearer in recent years, its potential application in retinal disease treatment remains largely unexplored.

This study aimed to investigate whether NG protects RPE cells from hypoxic stress injury, as well as its role in inhibiting neovascularization. To further assess its anti-neovascular properties, human umbilical vein endothelial cells (HUVECs) migration and lumen formation assays were performed. This study proposes a potential therapeutic strategy for BRVO treatment.

## 2. Materials and Methods

### 2.1. Reagents and Chemicals

Naringin (purity > 99%) was obtained from Shanghai Yuanye Bio-Technology Co., Ltd. (Shanghai, China). Fetal bovine serum (FBS), 0.05% trypsin, Dulbecco’s Modified Eagle Medium: Nutrient Mixture F-12 (DMEM/F-12), DMEM, and penicillin/streptomycin solutions were purchased from Gibco, Invitrogen (Carlsbad, CA, USA). Phosphate-buffered saline (PBS) and 3-(4,5-dimethyl thiazol-2-yl-)-2,5-diphenyl tetrazolium bromide (MTT) were obtained from Beijing Solarbio Science and Technology Co., Ltd. (Beijing, China). ROS, MDA, GSH, and SOD detection kits were purchased from Beyotime Biotechnology (Shanghai, China). The ELISA kits for IL-6, IL-1β, and TNF-α were purchased from Jianglai Biological (Shanghai, China), Shanghai. The basement membrane was purchased from Corning Incorporated (New York, NY, USA). The primary antibodies against NF-κB, ICAM-1, ACSL4, GPX4, HO-1, NQO-1, SLC7A11, and β-actin were all purchased from Abcam (Cambridge, UK).

### 2.2. Cell Cultures and Treatment

Human retinal pigment epithelial cells (ARPE-19) were purchased as frozen vials from Procell Life Science & Technology Co., Ltd. (Wuhan, China). The cells were cultured in DMEM/F-12 (supplemented with 10% FBS, 1% streptomycin/penicillin) at 37 °C in a humidified atmosphere containing 95% air and 5% CO_2_. Cells at 80–90% confluence were selected for subculture and subsequent experimentation. For CoCl_2_-induced apoptotic studies, the cells were cultured overnight in a serum-free medium, followed by exposure to CoCl_2_ (500 μM) for 24 h. HUVECs were purchased from iCell Bioscience Inc. (Shanghai, China). and cultured in DMEM supplemented with 10% (*v*/*v*) FBS and 1% penicillin/streptomycin at 37 °C in a humidified 5% CO_2_ incubator. The culture medium was refreshed every 1–2 days. All cell experiments were conducted using cells between the third and fifth passages.

Naringin, dissolved in DMSO, was stored at 4 °C. Based on our prior research, it was determined that a DMSO concentration below 0.1% does not induce cellular damage. Therefore, during the experimental conditions, this concentration was carefully maintained below the 0.1% threshold.

### 2.3. Cell Viability Assay and Morphology Examination

Cell viability was measured by MTT assay in the determination of the cytoprotective effect of NG on ARPE-19 cells. Overnight, the cells were plated in 96-well plates with a density of 4 × 10^3^ cells per well. After treatment with varying concentrations of NG (60–100 μM) for 24 h, CoCl_2_ (500 μM) was added, and treatment was continued for another 24 h. Cells without treatment were used as a control. After incubation, 100 μL of MTT working solution (0.5 mg/mL in new culture medium) was used to treat the cells at 37 °C for 4 h. Subsequently, 150 μL DMSO was added to each well to dissolve the water-insoluble formazan crystals formed. Cell viability was evaluated by measuring the absorbance of the formazan solutions at 590 nm using a microplate reader (Thermo, Multiskan, GO, USA). Morphological changes in ARPE-19 cells were observed and photographed under an inverted Olympus IX71 microscope (Olympus, Tokyo, Japan).

### 2.4. Transwell Assay

Cell migration was assessed with a 24-well transwell system containing 8 μm pore-sized filters without Matrigel. The HUVECs suspended in serum-free medium were added to the upper chambers, and cell culture medium was filled in the lower chambers. After 24 h of incubation, the filters were immobilized using 4% paraformaldehyde at room temperature. The migrated cells were subsequently stained using 0.1% crystal violet and observed under an inverted light microscope (Tokyo, Japan).

### 2.5. Tube Formation Assay

Tube formation assay was performed in Matrigel according to the manufacturer’s guidelines. After the Matrigel solidified, the cell suspension (1.5 × 10^5^ cells/well) was added, and the cells were treated with NG (60–100 μM) for 24 h. An inverted microscope was used to evaluate tube formation. Image-Pro Plus 6.0 software (Rockville, MD, USA) was used to determine the number of tubes.

### 2.6. Measurement of ROS Levels

DCFH-DA is a probe that freely passes through cell membranes and detects intracellular ROS. According to the manufacturer’s instructions, a diluted DCFH-DA fluorescent probe was added to the cells. The levels of ROS were determined in a CytoFLEX flow cytometer (BD Biosciences, Franklin Lakes, NJ, USA) and fluorescence microscopy (Olympus, Tokyo, Japan).

### 2.7. Measurement of GSH, MDA, and SOD Levels

The cells were lysed with ice-cold radio-immunoprecipitation assay (RIPA) buffer. The total protein concentrations of the lysate supernatants were determined using the Bradford protein assay kit (Biyuntian, Beijing, China). The levels of malondialdehyde (MDA), which is one of the important indicators, glutathione (GSH), an essential antioxidant substance, and superoxide dismutase (SOD), a key enzyme in antioxidant defense, in the supernatants were determined using their corresponding assay kits according to the manufacturers’ specifications. Data were calculated in terms of the protein concentration of each sample.

### 2.8. Western Blotting

The collected cells were lysed for 30 min on ice with RIPA buffer supplemented with protease inhibitors and then centrifuged at 10,000× *g* for 15 min, during which process the lysates were obtained. Following the determination of the protein concentration, all samples were subjected to boiling in loading buffer for 5 min in order to make the proteins denatured. Samples underwent SDS-PAGE and then were transferred onto PVDF membrane. The membranes, which were blocked with nonfat milk at room temperature for 2 h, were incubated with corresponding primary antibodies at 4 °C overnight. These membranes, which were then incubated with HRP-conjugated secondary antibody at room temperature for 2 h, were finally visualized by using chemiluminescence substrate. The intensity of all bands was measured by Image J (V1.51j8, NIH, Bethesda, MD, USA).

### 2.9. Quantitative Real-Time Polymerase Chain Reaction (qRT-PCR) Analysis

The methods of total RNA extraction and qRT-PCR have been described previously. [31]. Treat the ARPE-19 cells in the manner described previously. Then, the cells were collected, and the total RNA was isolated using TRIzol reagent (ThermoFisher, Waltham, MA, USA). Primer sequences of the targeted genes used in this study were as follows: HIF-1α(5′-CAGGAGCGAGACCCCACTAA-3′, forward; 5′-ATCACGCCACAGCTTTCCAG-3′, reverse) and VEGF(5′-TCACCAAGGCCAGCACAT-3′, forward; 5′-GGCTCCAGGGCATTAGACA-3′, reverse). All data were normalized to the mRNA expression level of GAPDH.

### 2.10. Statistics

Experiments were performed independently at least three times, and the results are expressed as mean ± standard deviation (SD). Statistical analysis was performed using GraphPad Prism 9.5 (GraphPad Software, Boston, MA, USA). The data were analyzed using Student’s *t*-test (statistical significance defined as *, *p* < 0.05; **, *p* < 0.01; ***, *p* < 0.001; ****, *p* < 0.0001) and one-way analysis of variance (ANOVA) followed by the Bonferroni/Dunn post hoc test (*p* < 0.05). Outliers were not identified or treated, and no transformations were applied to the data.

## 3. Results

### 3.1. Effects of CoCl_2_ and Naringin on Cell Viability

**Figure 1:**
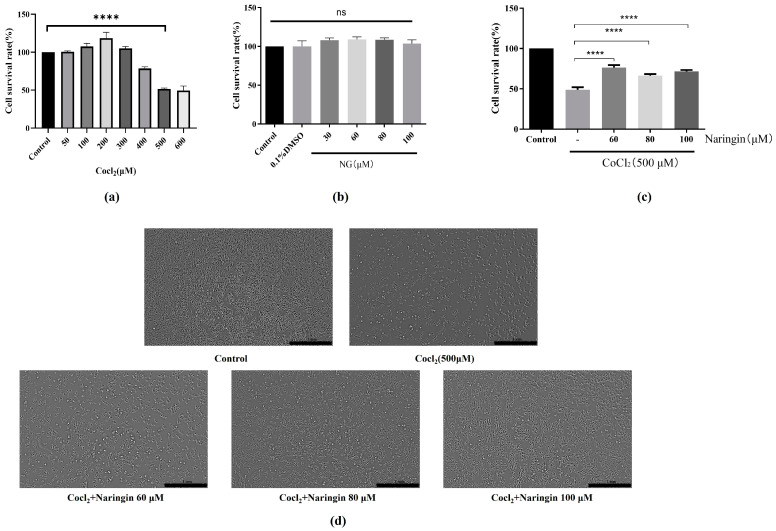
Naringin safeguarded RPE cells against damage induced by CoCl_2_. (**a**) ARPE-19 cells were first exposed to different concentrations of CoCl_2_ (50, 100, 200, 300, 400, 500, and 600 μM) for 24 h, and cell viability was determined with the MTT assay. (**b**) Effect of different concentrations of naringin on ARPE-19 cell viability. (**c**) Cytoprotective effect of naringin. ARPE-19 cells were pretreated with varying concentrations of naringin (60, 80, and 100 μM) for 24 h, and then CoCl_2_ (500 μM) was added, and the treatment was continued for 24 h. Cell viability was measured with the MTT assay. (**d**) Changes in the number of ARPE-19 cells were observed and presented. All the results are expressed as mean ± *SD*; ****, *p* < 0.0001; “ns” denotes non-significant (*p* ≥ 0.05).

To determine the toxic effects of CoCl_2_ on ARPE-19 cells, the cells were incubated with varying concentrations of CoCl_2_ (50~600 μM) for 24 h, and their viability was determined using the MTT assay. As shown in Figure 1a, CoCl_2_ significantly inhibited the viability of ARPE-19 cells in a dose-dependent manner. The IC_50_ value of CoCl_2_ in ARPE-19 cells after 24 h of exposure was determined to be 500 μM, which was subsequently used in further experiments. As shown in Figure 1b, naringin, within the tested concentration range, exhibited no toxicity to RPE cells. Thus, naringin concentrations of 60, 80, and 100 μM were selected for subsequent studies. To evaluate the cytoprotective effect of naringin, the cells were pretreated with increasing concentrations of naringin (60~100 μM) for 24 h before exposure to 500 μM CoCl_2_ for 24 h. Compared to the CoCl_2_-treated group, naringin pretreatment enhanced the viability of ARPE-19 cells in a dose-dependent manner (Figure 1c). Furthermore, the protective effect of naringin on CoCl_2_-treated ARPE-19 cells was further confirmed by monitoring changes in cell number (Figure 1d).

### 3.2. Naringin Pretreatment Attenuates Neovascularization in BRVO

**Figure 2:**
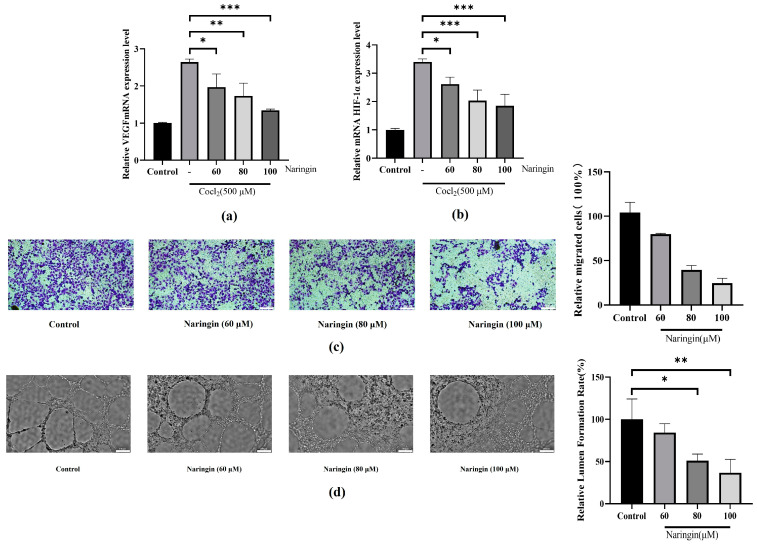
Naringin inhibits neovascularization. (**a**) Relative mRNA expression of HIF-1α. (**b**) Relative mRNA expression of VEGF. (**c**) Naringin inhibits the migration of HUVECs. (**d**) Naringin inhibits the tube formation of HUVECs. All the results are expressed as mean ± *SD*; *, *p* < 0.05; **, *p* < 0.01; ***, *p* < 0.001.

During BRVO pathology, hypoxia stimulates neovascularization by influencing the regulation of the HIF-1α/VEGF signaling in RPE cells, which has a dramatic effect on neovascularization [32]. ARPE-19 cells treated with CoCl_2_ exhibited a marked increase in HIF-1α and vascular endothelial growth factor (VEGF) secretion compared to the control group (Figure 2a,b). These findings suggest that naringin may exert a therapeutic effect by inhibiting neovascularization. To further explore its anti-neovascular properties, we examined the impact of naringin on tube formation in HUVECs. The experimental results demonstrated that naringin not only suppressed the migration ability of HUVECs (Figure 2c) but also significantly reduced their tube formation capacity (Figure 2d). In summary, naringin inhibits the HIF-1α/VEGF signaling pathway, suggesting that naringin has anti-neovascular effects. The experiments further demonstrate that naringin can inhibit migration and tube formation in HUVECs, potentially offering a new strategy for BRVO treatment.

### 3.3. Naringin Pretreatment Attenuates CoCl_2_-Induced Inflammation and Oxidative Stress in ARPE-19

**Figure 3:**
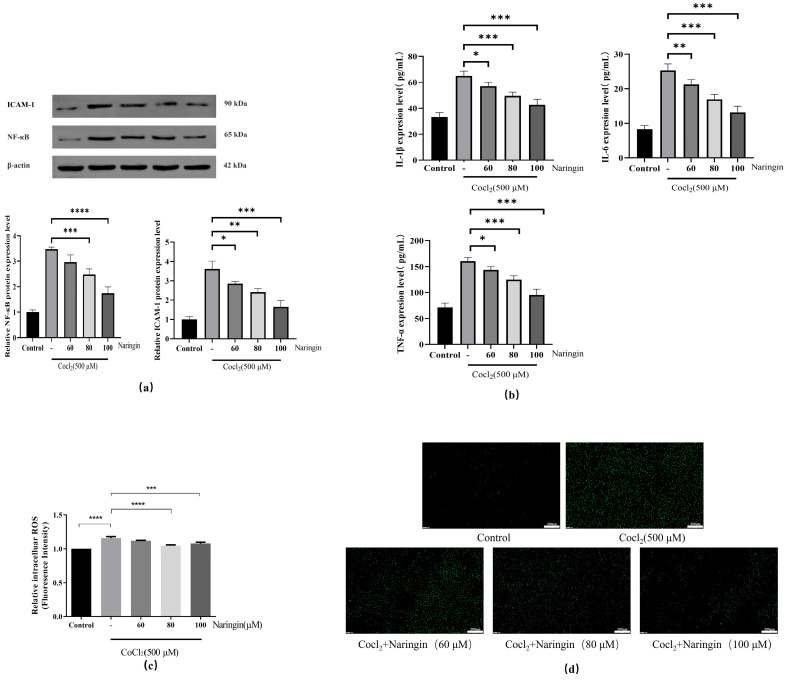
Naringin exerted an anti-apoptotic protective effect on CoCl_2_-induced RPE cells. (**a**) The expression levels of related regulatory proteins in the cell cycle were detected by Western blot assay. Actin-1 was used as an internal control. Data are presented as mean ± *SD* of three independent experiments. (**b**) The expression levels of IL-6, IL-1β, and TNF-α were measured by using an ELISA kit (Jianglai Bio, Shanghai, China). (**c**) Use the DCFH-DA assay to quantitatively measure the production of ROS. (**d**) Use the DCFH-DA assay to qualitatively measure the production of ROS. All the results are expressed as mean ± *SD*; *, *p* < 0.05; **, *p* < 0.01; ***, *p* < 0.001; ****, *p* < 0.0001.

We have investigated the inflammatory response of ARPE-19 cells under hypoxic conditions by measuring the level of inflammatory mediators such as NF-κB and ICAM-1. As shown in Figure 3a. The level of NF-κB and ICAM-1, which was increased by more than three times in CoCl_2_-induced ARPE-19 cells, was compared to that in control cells. Treatment with naringin inhibited the upregulation stimulated by hypoxic conditions. Meanwhile, ICAM-1, a recognized promoter of neovascularization and migration, further verifies the inhibitory effect of naringin on neovascularization [33,34]. Likewise, ELISA was utilized to detect the expression of inflammatory cytokines, including IL-6, IL-1β, and TNF-α, in ARPE-19 cells. The results demonstrated a significant increase in the expression of IL-6, IL-1β, and TNF-α in the model group compared with the control group (Figure 3b). DCFH-DA (2′,7′-dichlorodihydrofluorescein diacetate) is a cell-permeable probe widely used to detect intracellular ROS, with high sensitivity to hydrogen peroxide (H_2_O_2_) and some peroxynitrite derivatives. We found that compared with the control group, the expression of ROS in the model group was significantly increased; after treatment with naringin, the expression levels of ROS were significantly reduced (Figure 3c,d).

### 3.4. Naringin Inhibited CoCl_2_-Induced Ferroptosis of ARPE-19

**Figure 4:**
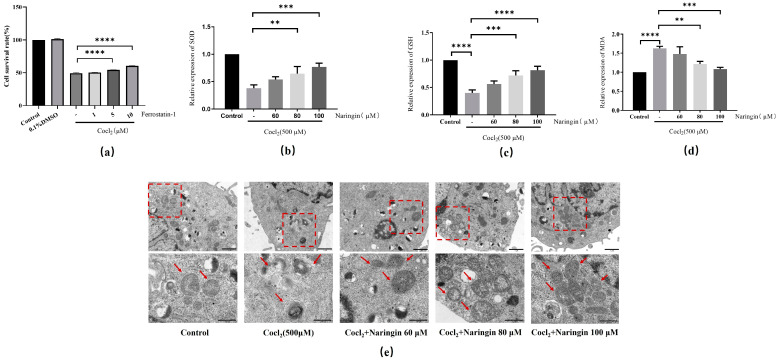
Naringin inhibits CoCl_2_-induced ferroptosis in ARPE-19 cells. (**a**) Effect of different concentrations of Ferrostatin-1 on ARPE-19 cell viability. (**b**) The levels of cellular SOD were determined. (**c**) The levels of cellular GSH were determined. (**d**) The levels of cellular MDA were determined. (**e**) TEM was performed to evaluate the microscopic changes in mitochondria. The structure in the red box is the mitochondrion and red arrows denote mitochondria. All the results are expressed as mean ± SD; **, *p* < 0.01; ***, *p* < 0.001; ****, *p* < 0.0001.

Naringin has been shown to exert a protective effect against anoxic damage [35,36]. However, whether naringin has a protective effect on CoCl_2_-induced ferroptosis remains unclear. Overproduction of ROS is one of the critical indicators of oxidative stress that triggers ferroptosis signals [37]. Meanwhile, the use of Ferrostatin-1 can significantly protect against CoCl_2_-induced cell death, confirming the abnormal activation of ferroptosis in CoCl_2_-incubated ARPE-19 cells (Figure 4a). Next, we evaluated the effect of naringin on ferroptosis in CoCl_2_-induced ARPE-19 cells. As demonstrated in Figure 4b,c, the decreased SOD and GSH expression caused by CoCl_2_ stimulation could be rescued by naringin. Moreover, naringin treatment inhibited the accumulation of MDA in the CoCl_2_-induced cells (Figure 4d). Considering that mitochondrial morphological changes are a hallmark of ferroptosis [38], an ultrastructural analysis by TEM showed that naringin significantly improved CoCl_2_-induced mitochondrial crista reduction and even disappearance and outer membrane rupture in ARPE-19 cells (Figure 4e).

### 3.5. Naringin Adjusted HO-1/GPX4 Axis to Improve Ferroptosis

**Figure 5:**
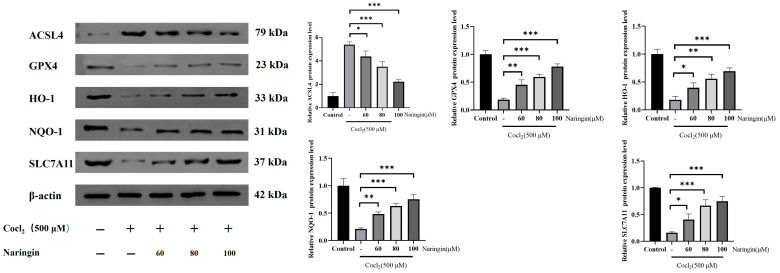
Naringin activated the SLC7A11 pathway in CoCl_2_-treated RPE cells. ARPE-19 cells were pretreated with or without naringin (60, 80, and 100 μM) for 24 h and then treated with 500 μM CoCl_2_ for 24 h. The expressions of ACSL4, GPX4, HO-1, NQO1, and SLC7A11 were measured with a Western blot assay. β-actin was used as an internal control for total protein. All the results are expressed as mean ± SD; *, *p* < 0.05; **, *p* < 0.01; ***, *p* < 0.001.

Under hypoxic conditions, the nuclear translocation of HIF-1α triggers the expression of antioxidant enzymes HO-1 and NQO1 [39]. The HO-1/GPX4 pathway is essential for regulating cellular oxidative stress resistance and ROS scavenging [40]. This signaling pathway is currently recognized as a key regulator of ferroptosis [41,42]. To assess ferroptosis-related protein expression, Western blot analysis was employed. CoCl_2_ treatment significantly reduced the protein expression of total GPX4, HO-1, NQO-1, and solute carrier family 7 member 11 protein (SLC7A11), while increasing Acyl-CoA synthetase long-chain family member 4 (ACSL4) protein levels. ACSL4 is a key regulator of lipid metabolism, closely linked to lipid peroxidation and ferroptosis [43]. These results suggest that ferroptosis occurred in CoCl_2_-incubated ARPE-19 cells (Figure 5). We propose that naringin modulates the HO-1/GPX4 axis to mitigate ferroptosis.

## 4. Discussion

The findings of this study demonstrate that naringin exerts protective effects on RPE cells under hypoxic conditions, primarily through the inhibition of ferroptosis. This is significant given the established role of ferroptosis in the pathogenesis of BRVO [44] and other retinal vascular diseases. The antioxidant properties of naringin likely contribute to its ability to alleviate oxidative stress, a key driver of ferroptosis [45]. Furthermore, our results suggest that naringin may also inhibit neovascularization, a critical factor in the progression of BRVO [46]. This dual mechanism of action—targeting both ferroptosis and neovascularization—positions naringin as a promising therapeutic candidate for BRVO treatment [47,48].

The observed reduction in hypoxia-induced cell death in RPE cells treated with naringin is consistent with previous studies demonstrating the compound’s protective effects in various oxidative stress and cell death models [49]. However, the specific pathways by which naringin inhibits ferroptosis in RPE cells have yet to be fully elucidated. Potential mechanisms may involve the modulation of lipid peroxidation, regulation of iron metabolism, or activation of antioxidant defense systems [50,51]. Future studies should focus on dissecting these pathways in greater detail to provide a more comprehensive understanding of naringin’s therapeutic potential.

Furthermore, naringin’s suppression of neovascularization suggests that it may also influence angiogenic signaling pathways, such as VEGF, which play a central role in BRVO [52]. This finding is particularly relevant considering the limitations of current anti-VEGF therapies, which, while effective, are associated with significant side effects and high treatment burdens. Naringin’s ability to target multiple pathological processes in BRVO could provide a more holistic treatment approach, potentially reducing the need for frequent intravitreal injections.

Therefore, this study provides compelling evidence for the therapeutic potential of naringin in BRVO, demonstrating its ability to protect RPE cells from hypoxia-induced ferroptosis and inhibit neovascularization. These findings emphasize the need for further research into the molecular mechanisms underlying naringin’s effects and its potential clinical applications. Future studies should also investigate the pharmacokinetics and safety profile of naringin to evaluate its viability as a therapeutic agent for BRVO and other retinal vascular diseases.

Firstly, cell viability assays demonstrated that naringin alleviates CoCl_2_-induced damage in ARPE-19 cells. Furthermore, naringin significantly reduces ROS production in RPE cells under hypoxic conditions, underscoring its protective role against hypoxic injury. While DCFH-DA detects a wide range of ROS, the observed fluorescence signal primarily reflects H_2_O_2_ accumulation, consistent with previous studies highlighting its key role in specific biological contexts, such as mitochondrial oxidative stress. While the DCFH-DA assay strongly suggests H_2_O_2_ as a major ROS, the specific contribution of H_2_O_2_ could be further validated using catalase treatment in future studies. These results highlight its potential as a therapeutic candidate for BRVO. Secondly, our study demonstrated that naringin effectively suppresses HIF-1α and VEGF mRNA expression in RPE cells while also inhibiting endothelial cell migration and tube formation. Under hypoxic conditions, RPE cells secrete large amounts of VEGF and other pro-neovascular factors [53]. VEGF is a pivotal driver of neovascularization, facilitating hypoxia-induced retinal vascular growth [54]. Retinal neovascularization extends along the retinal surface, heightening the risk of leakage and bleeding, which represent primary causes of blindness [55,56,57]. Therefore, our research suggests that naringin may inhibit retinal endothelial neovascularization associated with BRVO.

Subsequent findings revealed that pretreatment with naringin mitigated inflammation in hypoxia-damaged cells, as demonstrated by decreased levels of inflammatory factors, including IL-6, IL-1β, and TNF-α. Fer-1 treatment of ARPE-19 cells under hypoxic conditions significantly reduced cell death. We further observed that ARPE-19 cells exhibited ferroptosis-related pathological changes under hypoxia, including mitochondrial shrinkage and decreased inner mitochondrial cristae. Additionally, key markers of ferroptosis, including elevated MDA levels, along with reduced GSH and SOD levels, were also observed. Notably, pretreatment with naringin significantly ameliorated these cellular changes in ARPE-19 cells. Collectively, our results suggest that naringin protects RPE cells by downregulating inflammatory factors and inhibiting ferroptosis.

Finally, we conducted a preliminary investigation into the mechanism by which it inhibits cellular ferroptosis. HIF consists of a constitutive β-subunit and two oxygen-sensitive α-subunits (HIF-1α and HIF-2α). The activity of HIF is primarily regulated by oxygen-dependent proteolysis of the α-subunits [58,59,60,61]. HIF-1α plays a crucial role in metabolism, glucose transport, and cellular survival by modulating the expression of erythropoietin (EPO), VEGF, and HO-1 [62]. HO-1 serves as both a marker of ferroptosis and a critical antioxidant enzyme within the cellular defense system. Induction of HO-1 has been reported to exert beneficial effects on retinal degeneration [63]. For instance, the upregulation of HO-1 shields retinal endothelial cells from the stress caused by hyperglycemia [64]. NQO1 inhibits quinone redox cycling and ROS generation, thereby protecting cells from oxidative stress due to metabolic processes [65]. Solute carrier family 7 membrane 11 (SLC7A11) is an essential component of the system Xc-, and its inhibition can trigger ferroptosis [66]. GPX4 is a central regulator of ferroptosis, and its inactivation triggers ferroptosis through ROS accumulation resulting from lipid peroxidation [67]. ACSL4 is another crucial participant in lipid metabolism and ferroptosis [68]. Therefore, regulating the HO-1/GPX4 signaling pathway to inhibit lipid peroxidation and ferroptosis offers a viable therapeutic strategy. In this study, our results suggest that naringin inhibits CoCl_2_-induced ferroptosis and inflammation by regulating the HO-1/SLC7A11/GPX4 signaling pathway.

## 5. Conclusions

In summary, naringin alleviates neovascularization and mitigates hypoxic injury in RPE cells by regulating the HO-1/GPX4 signaling pathway, thereby suppressing ferroptosis. Our study suggests that ferroptosis may serve as a novel therapeutic target for BRVO, with naringin as a potential treatment option.

## Data Availability

The original contributions presented in the study are included in the article; further inquiries can be directed to the corresponding authors.
